# Rethinking neuropsychological test validity in dementia assessment: a critical review in the age of neuroimaging and digital markers

**DOI:** 10.3389/fnhum.2025.1578648

**Published:** 2025-10-08

**Authors:** Seyul Kwak

**Affiliations:** Department of Psychology, College of Social Sciences, Pusan National University, Pusan, Republic of Korea

**Keywords:** neuropsychological test, validity, dementia, criterion validity, construct validity

## Abstract

Neuropsychological tests are essential tools for evaluating dementia and related neurocognitive disorders, with their clinical utility determined mainly by their validity. This paper critically reviews the diverse evidence supporting the validity of neuropsychological tests in dementia assessment. Criterion validity is discussed in relation to the tests’ ability to predict clinical diagnoses and underlying brain pathology, with a focus on their sensitivity to functional impairments and progressive neuropathological changes. Construct validity is explored through the lens of cognitive processes underlying test performance, using evidence from correlation structures and experimental paradigms. Furthermore, the paper examines the impact of emerging digital technologies on the evaluation of neuropsychological test validity, highlighting contrasts with traditional validation methods. The review identifies discrepancies between different types of validity evidence, emphasizing the need to contextualize validity within specific clinical and research applications. By addressing the conceptual limitations and trade-offs between validation approaches, this study proposes a comprehensive framework for interpreting validity evidence. Ultimately, it offers theoretical and practical implications for enhancing the robustness of neuropsychological tests in clinical practice and research.

## Introduction

Neuropsychological assessment includes tools for evaluating individuals with suspected dementia. These assessments provide evidence to determine the presence of cognitive impairments, differentiate observed cognitive decline from normal aging, assess the severity of dementia, and identify the underlying pathology causing the deficits. Neuropsychological tests aim to measure cognitive abilities and detect deficits associated with specific brain structures and functions. Over the course of their long history, neuropsychologists have systematically gathered psychometric evidence to validate neuropsychological tests and ensure they serve their intended purpose.

Validity, commonly defined as the degree to which a test measures what it claims to measure, is a central consideration in the development and application of psychometric instruments (American Educational Research Association et al., 2014; Cronbach and Meehl, 1955). The type of validity sought for a test depends on its intended purpose and context, and the methods for establishing validity are similarly influenced by these objectives (Chen, 2018). Evidence supporting test validity is often categorized into two primary dimensions: (1) whether the test is practical and useful, and (2) what specific attributes the test possesses (Hughes, 2017). These dimensions correspond to criterion-based and construct-based validation approaches, representing two major frameworks of scientific evidence in test validation. Neuropsychological tests, like psychometric approaches in general, derive their validity from their clinical and research applications, with these two types of evidence forming the basis for validation.

While there is a broad consensus regarding the conceptual framework for validity, further discussion is required to address specific issues arising from contemporary science. Moreover, advancements in technological methodologies are challenging traditional clinical neuropsychological approaches, necessitating new validation methods. This review critically examines the various types of evidence supporting the validity of neuropsychological tests in the context of dementia and related neurocognitive disorders. It highlights the multiplicity of clinical criteria and construct-based evidence expected in these contexts and explores how traditional perspectives—such as empiricism and cognitivism—have been integrated into dementia assessment. Additionally, the review considers recent advancements in biomarker, neuroimaging, and digital marker research, examining how these developments have altered the landscape of validity evidence. Finally, it discusses potential discrepancies between different types of validity evidence, offering insights into their implications for clinical and research settings.

## Criterion validity of clinical outcome

Criterion validity involves evaluating whether a psychological test captures practical and meaningful real-world criteria, such as daily functioning or occupational adjustment, to validate its use as a tool for clinical diagnosis (Kane, 2001). This approach represents a foundational principle in clinical neuropsychology and has historically been one of the most widely utilized methods for test validation, often considered the “gold standard” in psychometric evaluation (Anastasi, 1950). Furthermore, empirical studies on the validity of neuropsychological test batteries frequently focus on this type of validation (Pawlowski et al., 2013). In the following sections, we explore criterion validity by examining its temporal dimensions (i.e., concurrent or predictive) and levels of analysis (i.e., clinical outcomes or brain pathology).

### Concurrent criterion validity

Concurrent criterion validity is the most rudimentary approach to criterion validation, where a psychological test is assessed against a criterion measured at the same point in time (American Educational Research Association et al., 2014). This type of validity relies on practical judgment criteria grounded in the specific and practical purposes of the test, allowing its utility to be verified without heavy reliance on theoretical constructs.

For many individuals undergoing neuropsychological assessments, two primary objectives emerge: (1) determining whether cognitive impairments are present due to a neurological condition, and (2) establishing whether these impairments currently affect the individual’s ability to perform daily activities. Clinical diagnoses summarize comprehensive judgments about changes in daily functioning caused by cognitive impairments, serving as a robust reference target for assessing the utility of testing tools. For example, the diagnostic criteria for dementia emphasize difficulties in independent daily functioning (McKhann et al., 2011), and neuropsychological measures of cognitive function are validated as reliably predicting these outcomes (Farias et al., 2003; Fields et al., 2010; Jefferson et al., 2006).

It should be noted, however, that two distinct aspects—neurological condition and functioning level—are intermingled within the criterion of clinical diagnosis, which in turn serve as separate benchmarks for validating neuropsychological tests. First, a primary criterion for test validity is the test’s ability to detect the organicity of impairment that indicates a neurological condition. In other words, the tests are deemed sensitive if the measured property responds to the presence of a biological cause of a clinical syndrome. Until the widespread availability of neuroimaging techniques in the 1980s, neuropsychological tests were predominantly used to identify the presence and track the location of brain injuries. At the time, validation relied on autopsy findings or neuroimaging as reference criteria, which formed the basis of tests as practical proxies for brain function measurement when direct assessment of neural damage was unavailable. For instance, a cluster of cognitive deficits frequently observed in patients with frontal lobe damage was referred to as “frontal lobe syndrome,” leading to the development of tests specifically designed to assess “frontal lobe functions” (Dubois et al., 2000; Malloy and Richardson, 1994). These traditional frameworks of the neuropsychological battery have been utilized to differentiate neurological conditions, which remain foundational to the development and composition of tests (Reitan and Wolfson, 2009).

Second, the functioning level implied in the clinical diagnosis also serves as another concurrent criterion for validity. For older adults with dementia, maintaining independent daily activities is directly linked to their quality of life and the burden experienced by caregivers. In cases where caregivers are unavailable to provide assessments of daily functioning, neuropsychological tests become a critical source of clinical inference. Tests measuring episodic memory and executive function consistently exhibit strong correlations with levels of daily functional capacity (Fields et al., 2010; Overdorp et al., 2016). Furthermore, neuropsychological tests often predict the degree of functional impairment more accurately than biomarkers associated with neurological conditions (Kwak et al., 2021b).

While the criterion of brain damage or functional impairment provides a foundational framework for evaluating test utility, this approach has inherent limitations, as the meaning of the criterion itself can be enumerated. For example, using “frontal lobe damage” as a criterion for test validation is problematic as an underspecified definition because individual patients exhibit substantial heterogeneity in the location, pattern, and extent of brain injuries. Consequently, tasks designed to measure specific brain functions (e.g., frontal lobe tests) are prone to misidentifying deficits in patients with frontal lobe damage (Demakis, 2004; Phillips, 2004).

Similarly, using clinical diagnoses as a criterion can raise other issues. While diagnostic categories often provide a convenient and practical reference for validating test utility, they may prioritize symptom description over the underlying disease entity or etiology. Because the characteristics of a disorder’s symptoms often result from complex and cumulative interactions of risk factors, the underlying disease stages, shared neuropathologies, and deficits in specific cognitive processes may remain unspecified. Even when a test demonstrates excellent diagnostic classification, its relationship to the biological or psychological construct it purports to measure may be unclear, leaving its interpretability vulnerable to alternative, unanticipated factors (Strauss and Smith, 2009). Consequently, while the evidence of diagnostic validity is suggestive of a test’s practical utility, the intrinsic validity of the criterion itself will remain largely unevaluated. For the advancement of operationally defining the diagnosis of dementia, researchers have advocated for separating clinical “disorders” from biologically defined “diseases” in diagnostic frameworks (Jack et al., 2024). However, this distinction has not yet been applied in the common practice of test validation.

Another limitation of concurrent validity is its reliance on criterion characteristics at a single time point, failing to account for changes over time. For instance, the DSM-5 diagnostic criteria for mild and major neurocognitive disorders emphasize objective cognitive decline as a core requirement. This reliance on test results during clinical diagnosis introduces the risk of criterion contamination, wherein the validity criterion itself is influenced by the test outcomes (Noel-Storr et al., 2014; Weuve et al., 2015). Such contamination risks exaggerate a test’s diagnostic utility through circular reasoning. When test validation studies lack pre-planned designs, information gathered from patients may inadvertently influence clinical criteria through indirect pathways. Even if the diagnosis is not explicitly contaminated by the leakage of target test results, as with mild cognitive impairment (MCI), diagnosis criteria derived from similar cognitive tests can still introduce inherently shared elements between the test under validation and the reference standard.

### Predictive criterion validity

One approach to maximizing the practical utility of criterion validity and addressing the limitations of concurrent validity is to evaluate predictive validity. Predictive validity assesses whether a psychological test provides information that can forecast future outcomes, distinguishing it from concurrent validity, which evaluates alignment with present criteria (American Educational Research Association et al., 2014). By using clinically meaningful benchmarks, predictive validity shares similarities with concurrent validity but emphasizes the test’s ability to predict future clinical outcomes. This approach mitigates issues of criterion contamination present in concurrent validation by prospectively evaluating whether a test can predict future clinical impairments.

Accumulating research highlights the significant role of neuropsychological tests in providing critical insights into the progression of dementia symptoms. Scores obtained through neuropsychological tests can predict the likelihood of dementia onset or rapid cognitive decline years in advance (Bäckman et al., 2005; Belleville et al., 2014; Chapman et al., 2011; Jang et al., 2017). While comprehensive neuropsychological tests are more time-intensive than screening tools, such as the Mini-Mental State Examination (MMSE), they offer superior accuracy in distinguishing mild cognitive impairment (MCI) patients who are at risk of developing dementia (Kim et al., 2017). Numerous studies further indicate that neuropsychological assessments enhance diagnostic accuracy and provide additional information about longitudinal changes in daily functioning, particularly for conditions of MCI and stroke (Donders, 2020). Thus, even within the same diagnostic category, neuropsychological tests contribute valuable explanatory power regarding future clinical outcomes.

It is also notable that repeated testing in follow-up assessments improves predictive validity by capturing changes over time. For example, one study has shown that older adults diagnosed with MCI and subsequently re-evaluated using MMSE or clinical rating scales showed dubiously low reversion rate from MCI to normal in the Alzheimer’s Disease Neuroimaging Initiative (ADNI) dataset (Thomas et al., 2019). In other words, a higher rate of MCI-to-Normal conversion implies the initial diagnosis of MCI was unreliable and less meaningful, which was the case when using a less valid cognitive test set. However, this artificial diagnostic bias could be adjusted by follow-up assessments incorporating neuropsychological tests, particularly those measuring episodic memory (Thomas et al., 2019). In another similar study, subtle cognitive changes observed in neuropsychological tests for 12 months have been shown to predict dementia onset with high accuracy over subsequent follow-ups (18–120 months) (Nation et al., 2019). These predictions often surpass the prognostic capabilities of major dementia biomarkers, highlighting the added value of neuropsychological tests. This series of studies demonstrates that repeated measurements of neuropsychological assessments enhance sensitivity to neurological changes and disease progression.

Both predictive and concurrent validity rely on clinically meaningful benchmarks, but the time point of assessment influences the validity evidence obtained. For instance, a study compared how a brief screening test and comprehensive tests correlate with current and future brain structural atrophy (Kim et al., 2017). The result showed that in patients already diagnosed with dementia or major neurocognitive disorders, MMSE captures the progression of neuropathology as effectively as comprehensive neuropsychological tests (Kim et al., 2017). Conversely, comprehensive neuropsychological batteries were uniquely accurate in detecting subtle future neuropathological changes in individuals with mild cognitive impairments. Furthermore, while hallmark clinical symptoms of Alzheimer’s disease (AD) often involve deficits in long-term memory, preclinical stages (a decade or more before onset) are frequently characterized by earlier changes in immediate memory (i.e., learning), processing speed, and executive functions (Amieva et al., 2014; Bilgel et al., 2014; Mortamais et al., 2017; Younes et al., 2019). In evaluating dementia risk, it is crucial to distinguish between concurrent clinical symptoms and preclinical signs, as these may represent different phenomena.

Another consideration when reviewing predictive and concurrent validity is that, in real clinical settings, objective tests interact in complex ways with subjective reports (patients’ subjective cognitive complaints, metamemory, and caregivers’ informant-rated observations of functioning), such that the apparent characteristics of validity may be modulated. Primarily, subjective measures of cognition contain predictive signals that are not captured by neuropsychological tests (Jessen et al., 2014; Lai, 2014; Perrotin et al., 2015). In addition, subjective reports can index contextual factors—such as anxiety/depression, insight, and environmental demands—that help recalibrate the clinical meaning of identical performance scores. This moderation can be observed explicitly within statistical models: considering neuropsychological tests in isolation may fail to capture clinical validity in the strict sense, whereas combining patient and informant reports with test scores can enhance the utility of certain measures (Kwak et al., 2023). Moreover, other studies show that baseline subjective cognitive complaints, even when only weakly related to concurrent objective cognitive performance, are associated with distinct long-term trajectories over extended follow-up (Morrison and Oliver, 2023). Accordingly, incorporating subjective reports alongside neuropsychological measures in predictive models leaves room to improve the predictive validity of existing test scores.

## Criterion validity of brain pathology

Distinguishing whether neural damage is present and whether cognitive problems reported by examinees are due to neurological conditions rather than psychiatric states has long been a fundamental goal of neuropsychological testing (Bilder, 2011). Many neuropsychological tests have been validated based on their sensitivity to detecting brain damage or disease (Golden et al., 1978) and can indirectly indicate the risk of associated pathologies in dementia evaluations. Significant neurological changes precede cognitive impairments of dementia, and neuropsychological tests included in dementia assessments are often designed to capture the neuropathological processes underlying observable behavioral symptoms sensitively (Fields et al., 2011; Mortamais et al., 2017).

Traditionally, the development of clinical neuropsychological tests has relied on the occurrence and localization of brain lesions as validation criteria. However, advances in neuroimaging and biomarker technologies now allow specific neuropathological features to serve as reference standards (Bilder and Reise, 2019). Neuropathological attributes detected through neuroimaging provide direct and indirect evidence of AD pathology, neurodegeneration in other types of dementia (e.g., frontotemporal dementia), focal brain injuries (e.g., stroke, infarction, traumatic injury), and cerebrovascular disease. This section introduces the characteristics of neuropathologies captured by different neuroimaging methods and their associations with neuropsychological test performance.

### Alzheimer’s disease pathology

Neuroimaging of AD pathology offers abundant information about the risk of rapidly progressing brain disease, even in cases without clinically observable symptoms. Positron emission tomography (PET) enables the quantification of Alzheimer’s-specific pathologies, such as amyloid-beta and tau protein deposits, and reduced glucose metabolism in the temporoparietal regions (Jack et al., 2013; Jagust, 2018). Structural magnetic resonance imaging (MRI) captures the extent to which these pathologies lead to neurodegeneration, such as atrophy in medial temporal lobe structures (Davatzikos et al., 2011; Fjell et al., 2009). These biomarkers often precede clinical diagnoses of dementia by 10–20 years and reveal heterogeneous clusters of dementia subtypes that cannot be easily distinguished based on clinical symptoms alone (Bateman et al., 2012; Dong et al., 2017). While the costliness of biomarkers limits their application in primary care settings, neuropsychological tests developed using biomarkers as reference criteria can improve diagnostic approaches (Glymour et al., 2018).

Structural and functional measurements of localized brain regions obtained through MRI also serve as neuropathological markers of AD. In the relatively advanced stages of Alzheimer’s pathology, structural neurodegeneration observed as global gray matter atrophy provides a prognosis for cognitive decline within a few years (Soldan et al., 2019). Neuropsychological performance reflects functional deficits caused by compromised neural resources that can no longer maintain existing cognitive functions, as well as the demand for additional compensatory neural resources. For example, longitudinal studies tracking brain structure and cognitive performance in older adults diagnosed with Alzheimer’s dementia or amnestic MCI over 1–5 years revealed that changes in neuropsychological test scores (e.g., dementia version of the Seoul Neuropsychological Screening Battery) were strongly correlated with cortical volume loss in individuals with MCI (Kim et al., 2017). By contrast, changes in screening tests (e.g., MMSE) only reflected structural changes in individuals already diagnosed with dementia. These findings demonstrate that precise neuropsychological tests, despite their higher time and cost demands, are more sensitive to severe neuropathological changes during early, asymptomatic stages than screening tools.

A systematic review demonstrated that AD pathology, particularly amyloid-beta accumulation, could be detected during preclinical stages, even in the absence of significant impairments in episodic memory, semantic memory, or executive function (Mortamais et al., 2017). Word list learning tasks have been shown to reflect structural changes in the medial temporal lobe, such as hippocampal and entorhinal cortex atrophy, and are strongly associated with amyloid-beta deposition. Poor performance on episodic memory tests corresponds to longitudinal degeneration of temporal cortical and hippocampal structures (Farrell et al., 2018; Fletcher et al., 2018; Hanseeuw et al., 2019).

Several works have validated neuropsychological tests against early-stage Alzheimer’s biomarkers. For instance, studies have explored the validity of an interference paradigm in word list learning tasks (where participants learn an interfering list of non-target items) to reflect Alzheimer’s pathology (E Curiel, 2013; Loewenstein et al., 2016). This paradigm showed sensitivity to medial temporal lobe volume changes and amyloid-beta accumulation. Composite factor scores combining multiple neuropsychological test measures of executive function have demonstrated stronger correlations with amyloid-beta, tau, and cortical atrophy biomarkers than individual test scores (Gibbons et al., 2012; Gross et al., 2012).

Additionally, composite scores from cognitive tests like word list and story recall, digit-symbol substitution test, and MMSE have been developed to detect cognitive decline in preclinical Alzheimer’s stages (Donohue et al., 2014; Hahn et al., 2020; Mormino et al., 2017). Although the precise underlying causes of cognitive deficits can often only be inferred circumstantially, AD pathology appears to reflect not only cortical-level degeneration but also the aggregated effects of white matter microstructural deterioration. This diffuse pathology may explain why tasks that demand the efficiency of inter-module interactions—rather than targeting a specific processing unit—consistently exhibit strong predictive validity for pathological progression. A prime example is the digit-symbol substitution test, which integrates processing speed, executive function, and motor coordination, thereby effectively capturing the widespread neural network disruptions characteristic of AD (Pichet Binette et al., 2021).

### Neurodegeneration

The mechanisms through which dementia-related pathological substances induce neurodegeneration vary between individuals, even among those with the same diagnosis (Noh et al., 2014; Tetreault et al., 2020; Vogel et al., 2021). The prominence of neurodegeneration in specific regions can influence the presentation of dementia subtypes (Tetreault et al., 2020). High-resolution neuroimaging enables the assessment of structural features (e.g., gray matter volume, cortical thickness) and functional attributes (e.g., hemodynamic responses) of affected brain regions. Differences in gray matter volume and functional activation across brain regions indicate variations in information-processing resources. Neuropsychological tests sensitive to regional neurodegeneration provide insights into the severity of neural damage and its impact on specific cognitive processes (Genon et al., 2018, 2022).

The assumption that neuropsychological tests reflect regional neural attributes may align with the traditional neuropsychological principle of double dissociation. According to this principle, selective deficits in specific cognitive tasks resulting from localized brain damage suggest that the tasks assess the unique functions of the localized brain regions (Teuber, 1955; Vaidya et al., 2019). With neuroimaging, this logic generalizes to subtle neuroimaging measures of signal intensity (i.e., voxel intensity) that are not overtly observed as lesions. For example, a series of works showed that long-term retention in word list tasks correlated with entorhinal cortex functionality, while visuospatial pattern recognition tasks correlated with dentate gyrus activity (Brickman et al., 2011, 2014). Similarly, recollection and recognition tasks showed double dissociation of correlating patterns, supporting their validity as measures of distinct medial temporal lobe structures (Argyropoulos et al., 2022; Yonelinas et al., 2022; Yonelinas et al., 2007). Additional research has revealed that learning and retention functions in episodic memory tasks reflect different neural patterns and gray matter volumes (Casaletto et al., 2017; Chang et al., 2010). Such findings underscore the selective sensitivity of neuropsychological measures to specific neural substrates and their utility in detecting diverse dementia pathologies during asymptomatic stages (Whitwell et al., 2009).

### White matter pathology and cerebrovascular disease

White matter refers to the neural tracts connecting distant brain regions, supported by microvascular networks that are particularly susceptible to dysfunction and damage (Prins and Scheltens, 2015). This structural characteristic can signify neurocognitive function distinct from gray matter morphology or functional activation. Vascular impairments can lead to damage in adjacent brain structures, a hallmark diagnostic feature of vascular dementia. However, white matter pathology is not exclusive to vascular dementia. Generalized neuropathological changes, including amyloid-beta and tau accumulation (characteristic of AD) and medial temporal lobe atrophy, also reflect potential risks associated with white matter degeneration (Boyle et al., 2013; Brickman et al., 2009; Lockhart and DeCarli, 2014). Structural brain imaging can identify white matter abnormalities, such as microbleeds or localized infarcts, which appear as hyperintense signals on imaging and provide evidence of the extent of structural neuropathology.

Imaging studies sensitive to white matter lesions have demonstrated that even in clinically asymptomatic older adults, subtle white matter changes and their spatial distribution are associated with increased risks of cognitive impairment. Neuropsychological tests measuring executive function and processing speed are particularly sensitive to white matter integrity and lesion burden (Birdsill et al., 2014; Debette and Markus, 2010; Habes et al., 2018; Hedden et al., 2012). Additionally, the specific location of white matter lesions correlates with declines in episodic memory performance (Brickman et al., 2018; Lockhart et al., 2012; Parks et al., 2011; Smith et al., 2011). Subdividing lesion patterns based on neural tract locations allows researchers to identify whether strategically significant white matter regions contribute to declines in neuropsychological performance (Brugulat-Serrat et al., 2019; Duering et al., 2013; Jiang et al., 2018).

Processing speed tests, rather than working memory tests, are specifically observed to be the most sensitive to cerebrovascular disease (Vasquez and Zakzanis, 2015). Sensitivity to white matter pathology can vary depending on task characteristics, such as whether time constraints are imposed, whether the task requires isolated modules, or whether processing speed tasks incorporate executive components (Lowry et al., 2021; Papp et al., 2014). These differences have led to the development of screening test combinations incorporating processing speed and motor function tasks to enhance diagnostic accuracy (Brookes et al., 2015; Kang et al., 2009).

Another neuropathology associated with dementia is stroke, which includes infarctions, ischemia, and hemorrhages. These events result in invasive brain damage, with behavioral deficits varying according to the locations of the lesions. Lesion mapping methods account for spatial variability in stroke-related damage at the group level (Sperber and Karnath, 2018). By superimposing brain lesion maps of patients exhibiting specific neuropsychological deficits, researchers can identify the brain regions most consistently associated with these impairments. Such analyses have validated neuropsychological tests designed to measure frontal lobe functions and factor-based intelligence scores by linking them to the affected brain regions (Gläscher et al., 2009, 2012). Accumulated lesion-mapping research suggests that highly localized functions, such as visual and motor skills, align closely with cortical regions, whereas tests for complex functions requiring extensive neural collaboration are more indicative of subcortical structures, white matter integrity, and disruptions in functional neural networks (Corbetta et al., 2015; Siegel et al., 2016; Sperber et al., 2022).

## Construct validity

In psychometric traditions, construct validation has been emphasized alongside criterion-based approaches (Campbell and Fiske, 1959; Cronbach and Meehl, 1955). Psychological constructs may emerge from social values, linguistic conventions, or practical utility (e.g., composite scores for socioeconomic status). However, from a realist perspective, validation seeks to reflect actual physical properties beyond the mere utility of the instrument (Fried, 2017). The process of validating a psychological test involves building theoretical foundations that extend beyond its practical application to represent meaningful psychological attributes (Cizek, 2016; Hughes, 2017).

Neuropsychological assessment incorporates cognitive tasks derived from experimental paradigms, applying theories of brain-behavior relationships. While criterion validity focuses on utility (e.g., clinical diagnosis or detection of neuropathology), construct validity requires a focus on ontology (whether the test measures an existing property) and epistemology (whether the property is measurable). Neuropsychological tests, like other psychological tests, must elucidate the cognitive abilities they assess and clarify the attributes contributing to individual differences in performance. Without such clarity, even tests with strong clinical discriminative power may struggle to provide an interpretation of how the prediction occurred (Strauss and Smith, 2009). For instance, MMSE, Montreal Cognitive Assessment (MoCA), and Alzheimer’s Disease Assessment Scale-Cognitive Subscale (ADAS-Cog) provide a summary score of multiple item modules that are difficult to apply detailed theoretical interpretation. Put differently, even when an individual is flagged as clinically at risk by an elevated total score, these instruments may lack a structured delineation of subconstructs within cognitive domains; consequently, the fine-grained interpretive meaning can vary with numerous extraneous factors (e.g., relative cognitive strengths, premorbid intellectual profile), making it difficult to attribute the observed global impairment to a specific underlying deficit. Despite the relative lack of evidence, construct validation is essential for explaining phenomena and understanding the cognitive processes underlying clinical outcomes (Jewsbury and Bowden, 2017; Pawlowski et al., 2013). This review explores construct validity by examining two key dimensions: (1) evidence from correlations among test performances (internal structure) and (2) evidence derived from analyses of cognitive processes.

### Evidence of internal structure

A common method of establishing construct validity involves examining the nomological network of correlations among variables (Embretson, 1998). This approach assumes that if unobservable attributes (e.g., cognitive constructs) exist, latent variables should causally influence observable behaviors (e.g., test performance), resulting in systematic covariation (Borsboom et al., 2004). Constructs are not directly observable; instead, their theoretical validity is inferred from expected relationships among multiple measures. For example, executive control functions are not represented by a single test but emerge from patterns of correlations across tasks involving different stimuli (e.g., visual, auditory, interfering elements), response modes (e.g., verbal, motor coordination), and task instructions (e.g., response targets, set-shifting). These structures of covariation inform theoretical frameworks about constructs (Friedman and Miyake, 2017; Miyake et al., 2000; Park et al., 2012). Within a covariance model, the construct of executive function can be subdivided into components such as inhibition, set-shifting, and updating, each of which can be measured through specific tasks.

Factor analysis of neuropsychological tests clarifies which measurements belong to specific constructs and the extent to which individual measures represent those constructs. First, a test’s representativeness determines whether it validly reflects a given construct (Brown, 2015). This enables researchers to infer which tests should be included in assessments of targeted cognitive domains, such as those used in diagnosing neurocognitive disorders or dementia, and to evaluate how well they represent those domains (Greenaway et al., 2009; Jones et al., 2024; Park et al., 2012). Inadequate representativeness results in low factor loadings and poor model fit, indicating that the hypothesized model structure fails to explain the observed covariance adequately. Comparing the fitness of different models helps determine whether tests broadly encompass constructs or require finer differentiation. Such cumulative evidence has informed diagnostic frameworks that identify behavioral impairments across distinct cognitive domains as defined in the criteria of neurocognitive disorder (Sachdev et al., 2014).

Factor models also guide the development of indices that enable interpretations of multidimensional measures in a neuropsychological battery (Jahng et al., 2015). For instance, the summed scores of weakly related measures form composites representing heterogeneous and broad constructs, whereas measures sharing latent factors yield theoretically coherent interpretations. Episodic memory, a composite cognitive ability, can be represented by indices combining multiple sensory modalities (i.e., auditory, visual) and memory processing stages (i.e., immediate short-term recall, delayed long-term recall) (Drozdick et al., 2011). Patterns of decline observed in these indices help interpret shared attributes of subtests and summarize refined information about constructs, thereby improving predictions of clinical outcomes and dementia pathology (Crane et al., 2012; Gibbons et al., 2012; Gross et al., 2014).

Despite its prevalent use in psychological test validation, expanding the interpretation of the internal structure approach can be challenging in the field of neuropsychological tests. One critical issue occurs in how much a given test can delineate finite units of construct by testing a structural model. When a certain test includes mixed cognitive elements, the measurements may not clearly align with distinct latent variables. For instance, fluency tests can be classified into varying domains, including executive function, processing speed, expressive language function, and semantic memory (Shao et al., 2014; Whiteside et al., 2016). Similarly, attentional tasks may appear to measure a unified cognitive domain but lack sufficient variance to support distinct subdomains empirically (Treviño et al., 2021). A common practice of factor analysis, which typically does not assume cross-loadings across latent constructs, may underspecify these aforementioned cases of multi-component nature. Moreover, the labeling of test scores can be critical in that how tasks are theoretically categorized can significantly alter interpretations of the number of impaired domains observed in examinees. The arbitrary categorization into cognitive domains can make clinical decision-making less reliable, as the severity, breadth, and underlying pathology of cognitive impairment directly affect whether deficits appear in single or multiple cognitive domains (Petersen et al., 2009).

Another issue arises from inconsistencies between traditional constructs and empirical findings, leading to reduced conceptual clarity. Higher-order factors often override the attributes of individual tests, particularly when analyses rely on normative populations (Bowden, 2004; Delis et al., 2003; Jacobson et al., 2004). For example, systematic variance in healthy populations may not align with expectations in patients with neuropathology, such as the hallmark long-term memory impairments seen in AD. Factor analyses within a normative population may obscure well-known distinctions between short-term and long-term memory structures, underestimating specific deficits caused by neuropathology (Holdnack et al., 2011). Only a few studies have explored measurement invariance across clinical status, which can explore whether latent factors diverge with the progression of neural dysfunction (Hayden et al., 2011).

The fundamental limitation of using internal structure as validity evidence lies in its inability to disentangle causal effects captured by correlations. From a perspective of realism, test scores are valid only if the target construct “exists” and the attributes of that construct causally generate the variance observed in test scores (Borsboom et al., 2004; Hughes, 2017). In some endeavors that suggest the role of working memory capacity in reasoning ability, studies were able to extend correlating evidence of structural equation modeling by experimentally manipulating additional task components (Engle et al., 1999; Harrison et al., 2015). However, correlations observed in cross-sectional data may reflect causal effects originating from a variety of mixed sources and a prolonged timespan other than underlying latent factors. For instance, the correlations between test performances and the effects of latent variables could arise from cumulative interactions associated with long-term cognitive development and aging processes (Kan et al., 2019; Kievit et al., 2017; Van Der Maas et al., 2017). It is important to acknowledge that these mechanisms can impose constraints on the theoretical extrapolation of causal interventions through latent variables. To extend the evidence of construct validity beyond a mere summary of information, it is important to note other alternative mechanisms that form the factor patterns of data.

### Evidence of convergent and discriminant validity

Convergent and discriminant validity are critical components of construct validation. These are often demonstrated by examining correlation patterns among theoretically related and unrelated measures. Convergent validity evidence is established when test scores show higher correlations with measures assumed to assess the same construct, while discriminant validity is evidenced by lower correlations with measures assessing different constructs (Campbell and Fiske, 1959; Stern et al., 2014; Westen and Rosenthal, 2003).

Specifically, tests within the same cognitive domain are theoretically expected to show stronger correlations with each other than with tests from other domains. However, the patterns of convergent and discriminant correlations often do not align with the traditional theoretical distinctions developed in neuropsychology based on lesion cases, and in practice, these domain-based correlations often lack clear distinctions (Dodrill, 1999).

One illustrative case is the shared variance between “hold” tests (which are resistant to neuropathology) and “no-hold” tests (which are sensitive to neuropathology), which often complicates interpretations despite their theoretical distinctions (Greenaway et al., 2009). Specifically, verbal comprehension ability is frequently used as a proxy for cognitive reserve. While this ability shows limited changes due to neuropathology and weak causal links to neurological conditions, it still exhibits notable correlations with neuropsychological measures known to be sensitive to pathology, such as delayed word recall (Siedlecki et al., 2009). Although factor structures and relative correlation patterns may be suggestive of distinctions between constructs, such evidence often fails to characterize the conceptual difference fully. This issue is particularly pronounced for tests that reflect both neuropathology sensitivity and premorbid cognitive ability.

The challenge also holds in the examination of predictive neuromarkers of neuropsychological tests. The finding showed that the multivariate model of brain functional connectivities was predictive of the overall domains of the test (Kwak et al., 2021a). In this way, the network-specific contribution to the test performance has been empirically tested. The finding suggests that meaningful brain-behavior predictability is largely comprised of shared intercorrelations and suggests the challenges in proposing discriminant validity. In other words, the brain’s functional characteristics predictive of a certain domain of the test are likely to predict all other test scores of other cognitive domains.

The lack of evidence of discriminant validity is a broader issue in psychometrics, where an overemphasis on convergent validity and proposing over-inclusive constructs paradoxically leads to blurred boundaries between constructs (Lilienfeld and Strother, 2020). When a wide range of measures is not available, researchers frequently rely on high correlations with homologous tests to claim convergent validity. This approach often relies on rejecting a less appropriate null hypothesis (“test will show no significant correlation with a target measure”). However, rigorous validation necessitates quantifying and testing whether the test demonstrates significantly larger correlations with theoretically aligned measures than with less related constructs (Westen and Rosenthal, 2003).

### Evidence of cognitive process

The foundation of construct validity lies in establishing theories about cognitive processes that underlie test performance (Brewer and Crano, 2014; Embretson, 1998). Experimental paradigms allow researchers to manipulate task stimuli, rules, and responses systematically, predicting behavioral differences and individual variations in cognitive processes. Such paradigms enable the development of tasks that capture specific cognitive impairments, extending theories about how certain pathological conditions affect behavior (Barch, 2005; Festa et al., 2005; Knight and Silverstein, 2001). For example, experimental tasks can measure how cognitive conflicts (e.g., conflicting stimuli and responses) slow reaction times. When systematic increases in interference conditions slow responses, the cognitive processes targeted by the task can be inferred. If individuals with specific neurological conditions exhibit amplified effects under experimental conditions, it suggests deficits in cognitive processes or resource allocation induced by the experimental manipulation.

Although the experimental task itself is rarely included in the clinical assessment battery, process analysis may form the theoretical basis for refining or modifying existing tests (Brickman et al., 2014; Loewenstein et al., 2016). For instance, tests developed through process-oriented approaches can identify the specific stages of language processing or episodic memory affected by brain damage. These qualitative measures not only clarify the nature of brain damage but also reveal disease risks not captured by composite scores (Delis et al., 1987; Goodglass and Kaplan, 1983). For example, differences in how individuals strategically organize information during memory encoding (e.g., semantic clustering) or intrusive errors during recall provide insights into specific deficits and align with neuropathology-based validity evidence (Kirchhoff et al., 2014; Thomas et al., 2020).

However, process-based approaches face challenges when divorced from other validation frameworks. By focusing on within-person variance, they often neglect inter-individual differences, creating a gap in practical utility. That is, systematic variance in behavior due to experimental conditions does not necessarily translate into meaningful individual differences, limiting the approach’s applicability for identifying cognitive attributes. This issue is particularly prevalent in tasks requiring complex cognitive integrations. For example, subtraction methods used to isolate specific cognitive processes (e.g., Stroop test: word reading versus color naming) yield stable experimental effects but often exhibit low psychometric reliability (Eisenberg et al., 2019; Enkavi et al., 2017). Measurement error, rather than targeted cognitive attributes, often accounts for most of the observed variance (Hedge et al., 2017).

## Disagreement of validity evidence

### Disagreements within criterion validity

The diversity of validity evidence extends beyond conceptual differences to include the critical issue of potential inconsistencies among different forms of evidence. For example, individuals with increased measures of cognitive reserve may reach functional thresholds at a more delayed time point in later life. This in turn leads to the observation that tests with concurrent criterion validity in predicting clinical outcomes like functional impairment in dementia (Tucker-Drob, 2019). However, these measures do not directly correspond to the progression of AD pathology. In other words, there is a misalignment between two criterion variables: functional impairment and the presence of neuropathology. Such disagreements are reflected in ongoing debates about the operational definitions of AD as a biological disorder versus a clinical syndrome (Jack et al., 2024; Petersen et al., 2021). As biomarkers become increasingly accessible, the incompatibility between clinical manifestations and biological markers has become more apparent. This necessitates a clearer delineation of the operational criteria that neuropsychological tests are validated against (Hazan et al., 2024).

In the case of the criterion within brain pathology, inconsistencies arise due to differences in the specific characteristics of pathology. For instance, while neuropsychological batteries used in dementia assessments often predict the severity and progression of dementia, their predictive validity for differentiating subtypes (e.g., Alzheimer’s vs. vascular dementia) may not align with each other. Certain subtests, such as constructional praxis, provide strong discriminative information about dementia subtypes but are less sensitive to overall clinical severity (Kwak et al., 2023). These discrepancies may arise from the qualitative differences between focal brain lesions specified from a classical validation design (i.e., clinical case–control) and diffuse disruptions seen in dementia-related neuropathology. Focal lesions, such as those caused by stroke, produce specific behavioral impairments tied to discrete brain regions. In contrast, the distributed nature of neuropathological changes in dementia may limit the utility of narrowly focused cognitive tasks in capturing widespread neural deficits.

The progressive and staged nature of dementia also complicates the validation target. Sensitivity to cognitive domains may vary across disease stages, even within the same dementia type (Kim et al., 2024; Levin et al., 2021). For example, delayed recall functions, which reflect retention processes, often show pronounced declines in later stages of dementia but may not fully capture changes during the preclinical phase. By contrast, executive function tasks such as the Digit Symbol Substitution Test can provide additional information about early-stage changes that memory tasks alone fail to explain.

### Disagreements between construct and criterion validation

The mismatch between construct clarity and criterion validity often leads to a tradeoff in the neuropsychological test aim. Construct validity involves refining experimental conditions to isolate specific cognitive processes. For example, tasks may be designed to manipulate stimuli and response rules parametrically or subtract baseline conditions to enhance the precision of targeted constructs. By controlling extraneous variance, test developers aim to hone the clarity of the constructs being measured. However, this refinement may not translate into stronger ecological validity or predictive power for everyday functioning. Paradoxically, tasks designed to measure specific cognitive processes often demonstrate weaker criterion validity for predicting real-life outcomes (Gold et al., 2012; Sbordone, 1997). This has been termed as two approaches of conceptual framework: verisimilitude and veridicality (Chaytor and Schmitter-Edgecombe, 2003). Verisimilitude is the degree to which the cognitive demands of a test theoretically resemble the cognitive demands in the everyday environment, whereas veridicality refers to the degree to which existing tests are empirically related to measures of everyday functioning (Franzen and Wilhelm, 1996).

Specifically, a nuanced tradeoff can be found in the case of cognitive ability tests. Tasks with more clear specification of the cognitive construct may provide less criterion-relevant information. For example, while general cognitive ability strongly predicts clinical diagnoses, specific abilities reflected in each cognitive domain often contribute a limited amount of incremental information (Breit et al., 2024). Likewise, the verbal comprehension index from intelligence tests is reflective of cumulative developmental experiences rather than an explicit sampling of cognitive processes. Despite their construct ambiguity and unspecifiable process, these measures often show robust predictive validity for clinical impairment (Ackerman, 2022; Royall and Palmer, 2012).

This trade-off is also observed in other performance-based tests. The clock drawing test, for instance, requires multiple domains of the process—executive function, visuospatial construction, motor skills, and semantic knowledge—but inevitably lacks detailed specification of how cognitive processes contribute to the observed functioning. Nonetheless, it remains effective in detecting neurocognitive impairments due to dementia (Tsoi et al., 2015). Similarly, real-life tasks like the Multiple Errands Test (MET) have demonstrated strong psychometric properties but remain uncertain about how specific cognitive domains, such as executive function, constitute the overall performance (Rotenberg et al., 2020).

The challenges in the divergence between construct validity and ecologically oriented criterion validity may become more prominent in tasks of complexity and multiplicity of components. It is more difficult to standardize and control experimental structure in tasks requiring a certain time for reasoning and mobilizing strategies. Some of the works have designed ways to assess by determining the major everyday cognitive ability required by the environment (Chaytor and Schmitter-Edgecombe, 2003). High-order cognitive abilities, however, often involve heterogeneous strategies and functional brain activity patterns, even among individuals with similar test scores (Barulli et al., 2013; Seghier and Price, 2018). This tradeoff reflects the adaptive and flexible nature of real-life problem-solving, which depends on the dynamic integration of cognitive processes rather than the efficiency of a single isolated process. Consequently, it would be less surprising to find that cognitive tasks emphasizing ecological validity, even at the expense of construct refinement, may hold greater utility in dementia evaluations (Chaytor and Schmitter-Edgecombe, 2003; García-Betances et al., 2015; Howett et al., 2019; Kessels, 2019).

Choices of scoring practices also regard the tradeoff between clarity of construct and predictability of criterion. Neuropsychological tests typically exhibit positive manifolds (i.e., intercorrelations) among subtests, enabling the aggregation of composite scores based on shared variance (Agelink van Rentergem et al., 2020). A total score across multiple cognitive domains or summing subtests as a composite score often shows robust predictive validity for clinical criteria, providing sensitive and accurate information about impairments (Chandler et al., 2005; Fields et al., 2010; Wolfsgruber et al., 2014). However, this expansion in criterion validity may come at the expense of construct clarity. Neuropsychological batteries tend to have lower internal consistency than factor-based ability tests, such as intelligence tests, indicating limited homogeneity of constructs underlying total scores (Jones et al., 2024; Kiselica et al., 2020). This suggests that improved criterion validity may be partially achieved by compromising the conceptual coherence of constructs.

### Digital markers: redefining validation standards

Traditional neuropsychological assessments have relied on a concise set of scores to evaluate predictive validity and construct-based interpretations. However, advances in digital markers introduce challenges that require alternative validation approaches (Kumar et al., 2021). First, machine learning (ML) algorithms no longer rely on the linear combinations of individual scores. For instance, adding subtests in a battery may fail to improve criterion validity under linear models, whereas such addition yields significant gains in predictive accuracy with nonlinear ML models (Kwak et al., 2022). Such superiority of nonlinear algorithms is well-documented across various predictive domains (Schulz et al., 2020). This indicates that the validity of individual test scores contributing to diagnostic information can vary depending on the specific ML algorithm employed.

Predictive modeling becomes increasingly critical as the dimensionality of available information grows (Yarkoni and Westfall, 2017). Digital phenotyping involves high-dimensional data from device logs, daily activity metrics, wearable sensors, and voice features (Palliya Guruge et al., 2021). Although the digital data typically lacks simple summary measures, they provide sparsely distributed predictive information across multiple features which overall contributes to approximating neurocognitive function to some extent (Hackett and Giovannetti, 2022; Harris et al., 2024; Holmes et al., 2022). From the perspective of validity evidence, a principal challenge is that these approaches yield such a profusion of variables that individual verification becomes infeasible, thereby complicating construct-based interpretations of criterion validity. Moreover, in such contexts, the predictive power of individual predictor variables and the utility of the measurement modality may lead to divergent inferences. For example, even if dozens of voice features exhibit poor criterion validity and one of numerous line-drawing features demonstrate excellent criterion validity, the feature set may nevertheless achieve superior aggregate criterion validity when the assessment module is evaluated at the whole set level. A “simulated lesion” analytic strategy—designed to assess the overall contribution of a specific modality set—can quantify both how many assessment modules (e.g., voice and drawing module) should be included and the magnitude of their practical usefulness with respect to the criterion (Hahn et al., 2017; Kohoutová et al., 2020).

## Conclusion

This review examined the contemporary issues underlying the criteria for deeming neuropsychological tests as “valid” for dementia assessment. It also explored how advances in neuroimaging and biomarker research necessitate changes from traditional validation processes. Unlike the conventional approach of validating the correspondence between constructs and criteria through studies on clinical cases of brain damage, the proliferation of biomarkers and neuroimaging technologies—capable of identifying diverse attributes of brain pathology (e.g., regional patterns, functional deficits, and types of pathology)—has broadened the validity criteria supporting the utility of neuropsychological tests. Additionally, this review underscored the need to re-examine the varied and assumption-specific nature of evidence for validity.

It is emphasized that neuropsychological tests may fail to meet various validity criteria in a consistent manner, not necessarily due to their inherent limitations of validity, but potentially because of fundamental discordance of the conceptual status of validity. This discussion highlights that validity is not monolithic and that different tools may meet distinct criteria depending on their specific constructs and intended applications.

**Figure 1:**
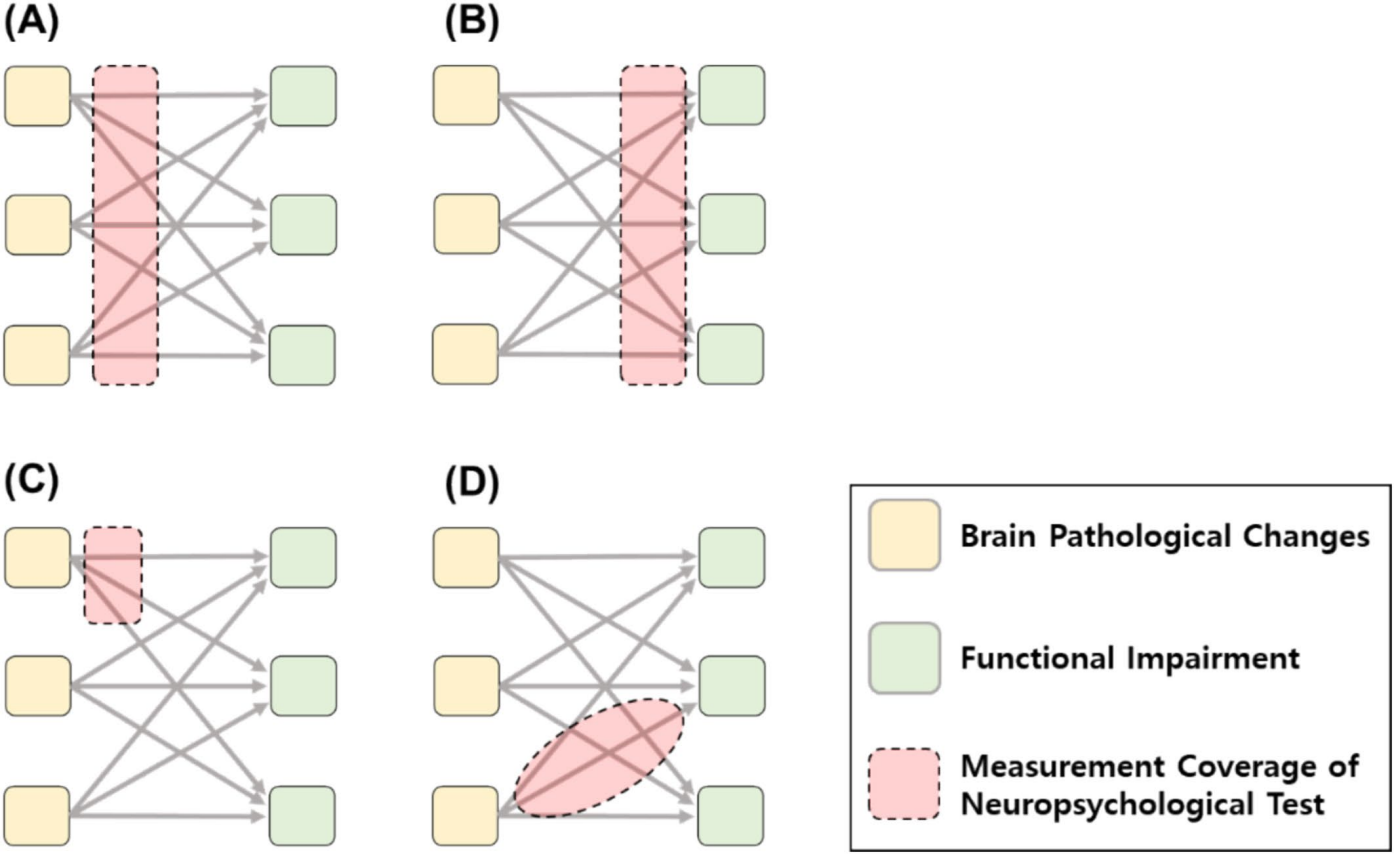
Various validity positions and goals of measurement coverage of neuropsychological test. **(A)** Measurement coverage sensitive to brain pathology criterion. **(B)** Measurement coverage sensitive to functional impairment criterion. **(C)** Measurement coverage to specific neuropathology changes. **(D)** Measurement coverage corresponding to specific construct.

The accompanying figure illustrates the conceptual framework within which the validity of neuropsychological tests for dementia evaluation is established. Depending on the test, the domain of evidence may vary, influenced by the relative strength of construct and criterion validity, as well as the scope and specificity of measurement. For instance, tests may differ in their measurement emphasis on either sensitivity to pathology underlying the disease (Figure 1A), ecological validity and sensitivity to functional outcomes (Figure 1B), capacity to characterize unique attributes of brain pathology (Figure 1C), or their ability to capture specific processing units and explain the continuum from disease to disorder with robust construct validity (Figure 1D).

This review also invites a more refined generalization of the evidence-based assessment approach into the practice of neuropsychological test validation (Hunsley and Mash, 2007). However, some neuropsychological test cases can be challenged by fundamentally disagreeing validity evidence and trade-offs between the aims of evidence, where the primacy of one type of evidence may compromise another. In other words, the validity of neuropsychological tools needs to be evaluated under their specific purposes and contexts, rather than seeking straightforward alignment of validity evidence as a parallel criterion.

Ultimately, validity evidence reflects not only the inherent soundness of an instrument’s internal logic but also its dependence on external contexts. Researchers and practitioners are encouraged to explicitly articulate the specific contexts in which validity evidence is established, enabling more precise interpretations of such evidence. Conversely, usage guidelines can be formulated under context-specific validity constraints—for example, by explicitly noting that validity has not yet been demonstrated in certain contexts, or by directing that interpretation be expressly deferred for particular subconstructs within a cognitive domain. This contextualized perspective of the validation process will be critical for advancing the clarity and utility of validity claims in neuropsychological assessment.
